# Infant skin-cleansing product versus water: A pilot randomized, assessor-blinded controlled trial

**DOI:** 10.1186/1471-2431-11-35

**Published:** 2011-05-13

**Authors:** Tina Lavender, Carol Bedwell, Ediri O'Brien, Michael J Cork, Mark Turner, Anna Hart

**Affiliations:** 1 School of Nursing, Midwifery and Social Work, The University of Manchester, Manchester, UK; 2 School of Medicine and Biomedical Sciences, University of Sheffield, UK; 3 School of Reproductive and Developmental Medicine, University of Liverpool/Liverpool Women's Hospital, Liverpool, UK; 4 Division of Medicine, Lancaster University, Lancaster, UK

## Abstract

**Background:**

The vulnerability of newborn babies' skin creates the potential for a number of skin problems. Despite this, there remains a dearth of good quality evidence to inform practice. Published studies comparing water with a skin-cleansing product have not provided adequate data to inform an adequately powered trial. Nor have they distinguished between babies with and without a predisposition to atopic eczema. We conducted a pilot study as a prequel to designing an optimum trial to investigate whether bathing with a specific cleansing product is superior to bathing with water alone. The aims were to produce baseline data which would inform decisions for the main trial design (i.e. population, primary outcome, sample size calculation) and to optimize the robustness of trial processes within the study setting.

**Methods:**

100 healthy, full term neonates aged <24 hours were randomly assigned to bathing with water and cotton wool (W) or with a cleaning product (CP). A minimum of bathing 3 times per week was advocated. Groups were stratified according to family history of atopic eczema. Transepidermal water loss (TEWL), stratum corneum hydration and skin surface pH were measured within 24 hours of birth and at 4 and 8 weeks post birth. Measurements were taken on the thigh, forearm and abdomen. Women also completed questionnaires and diaries to record bathing practices and medical treatments.

**Results:**

Forty nine babies were randomized to cleansing product, 51 to water. The 95% confidence intervals (CI) for the average TEWL measurement at each time point were: whole sample at baseline: 10.8 g/m2/h to 11.7 g/m2/h; CP group 4 weeks: 10.9 g/m2/h to 13.3 g/m2/h; 8 weeks: 11.4 g/m2/h to 12.9 g/m2/h; W group 4 weeks:10.9 g/m2/h to 12.2 g/m2/h; 8 weeks: 11.4 g/m2/h to 12.9 g/m2/h.

**Conclusion:**

This pilot study provided valuable baseline data and important information on trial processes. The decision to proceed with a superiority trial, for example, was inconsistent with our data; therefore a non-inferiority trial is recommended.

**Trial registration:**

ISRCTN72285670

## Background

The main role of the baby's skin is to provide a barrier which prevents infection, the loss of water from the body, and penetration of irritants and allergens. These functions depend on the maintenance of skin integrity and pH balance. Babies are born with a pH of 6.4 which reduces over three to four days to around 4.9 [1]. A baby's skin has a less developed epidermal barrier than adults and thus is more prone to damage; recent research suggests that the stratum corneum of infants becomes 'adult-like' only after one year of life [2]. The immaturity of babies' skin creates the potential for a number of skin problems, including atopic eczema, infant Candida, cradle cap, baby acne and napkin dermatitis [3]. These problems emphasize the importance of appropriate skin cleansing routines.

The guidelines, '*Routine postnatal care for women and their babies' *[4], in the UK, recommend that cleansing agents added to bathwater should be avoided in the early postnatal period. In contrast, The American Association of Women's Health, Obstetrics and Neonatal Nursing (AWHONN) [5] produced clinical guidelines that recommend the use of warm tap water for routine bathing with the option to use mild cleansers that have a neutral pH (5.5-7.0). However, there is a lack of evidence on which to inform practice for the term newborn baby. A survey of maternity units in the North West of England [6] reported that a wide range of products were used by women. Moreover, a systematic review of skin care regimes, in the well term newborn, revealed no prospective trials that met the authors' inclusion criteria [7]. As such, there are no UK evidence-based guidelines about neonatal skin care [8].

The Royal College of Midwives [9] called for further research in this area. A recent European round-table of Dermatologists also acknowledged the dearth of evidence for skin care provision within 6 weeks of birth [10]. The absence of randomized controlled trials comparing different skin cleansing routines is an important issue because of the readiness to use wash products among mothers [11].

Water is the basic component of any cleansing routine. In many countries, despite the lack of strong evidence in one direction or the other, water alone has been considered the least harmful of all alternatives [4]. However, water may not be the optimal skin cleanser for newborns. The buffering capacity of water is being questioned, as it might increase skin pH; after washing with water the skin surface pH may rise from 5.5 to 7.5. This brings the pH to a level that maximizes the activity of the skin proteases and therefore enhances skin barrier breakdown [12]. The other problem with water alone is that it is a poor cleanser as it does not remove fat-soluble substances such as feces and sebum [13]. On the extreme, over-exposure to water leads to higher trans-epidermal water loss (TEWL) and a weakened skin barrier [12]. An appropriately formulated cleansing product may reduce these potential problems but would need to be carefully evaluated.

Prior to the commencement of our study (in 2008), we identified only two small trials that compared baby bathing with a cleansing product to water. Both were available in abstract form only, so our assessment of methodology and interpretation of findings was necessarily limited [14,15]. Following a small-scale study involving 57 infants, Garcia Bartels' conclusion was that skin barrier development of term newborns was not adversely effected by bathing with a mild detergent cleansing product. Galzote [14], using a different wash product to Bartels [15], found that skin dryness was reported more often in the 'water only' arm. These trials were not large enough to provide definitive guidance.

One concern during skin care is atopic eczema. This is a disease that arises as a result of the interaction of environmental factors (such as harsh soap & detergents) with variants in several genes [16,17]. Atopic eczema starts as a weakness of the skin barrier [16-22]. This breaks down allowing allergens to penetrate the skin and interact with the immune system. Some of the damage is caused by enzymes in the skin; proteases. Proteases are pH sensitive enzymes with optimal activity at 7.5 to 8.0 [20,21]. Harsh soap and detergent raise the pH of the skin to within this range thereby increasing the protease activity in the skin and potentially leading to severe skin barrier breakdown. Washing with a detergent which can damage and break down the skin barrier may lead to an atopic flare in susceptible infants. This may be important in bathing practices for newborn babies, but this possibility has not been accounted for in previous work.

As there was limited previous research in this area and the available studies did not report key details of methodology, careful preparation was required for an adequately powered investigation. We therefore conducted a qualitative, exploratory study [11] to gauge support for a trial of bathing practices for term newborn babies, in the UK. The results highlighted the inconsistencies in information provided to parents and in current newborn bathing practices. It also demonstrated that health professionals and parents were likely to support a trial.

Therefore, we conducted a pilot randomized controlled trial to compare a skin cleansing agent (specifically formulated for use on newborn skin), to water. We hypothesized that an optimally formulated infant skin-cleansing product improves skin barrier function (measured by TEWL) in newborn babies when compared with bathing with water and cotton wool.

The pilot was designed to address the following uncertainties in the design of the full study: the practicability of using TEWL on newborn babies; the best outcome to use (TEWL, pH or hydrometer), the best locations to use (arm, leg or abdomen), the optimal time point for measurement of the primary outcome and the value of key parameters in the sample size calculation (the magnitude of difference that would be important to detect between the two groups and the precision of our measurements).

## Methods

### Study site and Population

A randomized study was conducted from November 2008 to November 2009 in a teaching hospital in the North West of England, where more than 8000 babies are born annually. Babies were included if they were born at 37 weeks gestation or more and were in good general health (determined by the investigator). Excluded babies were those admitted to the neonatal unit; having phototherapy; limb defects; non-traumatic impairment of epidermal integrity or evidence of skin disorder at first visit. For the purposes of this study, the following normal variations were not considered skin disorders; erythema neonatorum, erythema toxicum and milia. Babies were also excluded if participating in another clinical trial.

We set out to recruit a sample of babies with a family history of atopic eczema (*n *= 30) and a sample of babies who did not (*n *= 50). We believed that any effects were likely to be more pronounced in infants with a family history of atopic eczema and therefore we accounted for this in the design of the trial. These numbers were deemed to be sufficient to explore the nature and sizes of differences in outcomes and to estimate the standard deviations for each population.

The trial was approved by the Cheshire Research Ethics Committee (09/H1017/3).

### Recruitment and randomization

All potentially eligible women were supplied with study information in the antenatal period and given time to consider participating. Willing participants were invited to complete a self administered questionnaire; this enabled us to screen for those with and without a family history of atopic eczema. The definition of "family history of atopic eczema" was "at least one of father, mother, or sibling, who has had a medical-diagnosis of atopic eczema and who has had topical steroid treatment". We considered this to be the simplest way of identifying babies with a predisposition for atopic eczema.

In the postnatal period a research midwife approached women who had completed the questionnaire and requested consent for their baby to participate in the trial. Consenting women were randomized to the experimental or control arm within 24 hours of giving birth and prior to their baby being given his/her first bath. Randomization was stratified according to whether or not the baby fulfilled the definition of a family history of atopic eczema. Blocked randomization was by sequentially numbered sealed opaque envelopes held in the Trust R&D Department. The randomization sequence was computer generated.

### Intervention

Babies were randomized to be bathed in water only or bathed with the baby wash product. The wash product was the commercially available Johnson's^® ^*baby top- to-toe™ wash *(Johnson & Johnson Consumer Companies, Inc.). This wash is a soap-free liquid cleanser specifically designed for newborns' skin. It is sodium lauryl sulphate free and consists of a proprietary blend of non-ionic and amphoteric surfactants that, when combined, result in large, gentle cleansing micelles. The formula contains only strictly necessary levels of well-tolerated preservatives and a very low level of fragrance; it is pH adjusted (around 5.5) and hypoallergenic. The INCI list comprised Aqua, Coco-Glucoside, Cocamidopropyl Betaine, Citric Acid, Acrylates/C10-30 Alkyl Acrylate Crosspolymer, Sodium Chloride, Glyceril Oleate, p-Anisic Acid, Sodium Hydroxide, Phenoxyethanol, Sodium Benzoate, Parfum.

All participating mothers were given a demonstration bath by a Health Care assistant who had been instructed on the appropriate advice. For those allocated to the water only (control) arm, parents were not provided with any products and were advised to bathe their baby with water and cotton wool only. For those allocated to the wash product (experimental) arm, parents were provided with sufficient baby wash and advised to use the product as per instructions.

All participating parents were supplied with written guidance on baby bathing. These instructions included guidance on regularity of bathing and the non use of other products, e.g. oils, sponges, flannels and baby wipes. Participating women were requested to bathe their baby a minimum of 3 times per week. The number of times babies were bathed was recorded by the women. They were also instructed to avoid any rubbing of the baby's skin and requested not to use any additional products.

### Assessment of trial outcomes

All measurements were taken by researchers who were unaware of treatment allocation. Measures were repeated to check for intra-rater reliability. At the outset we had intended to conduct all assessments in a controlled environment within the hospital setting. All baseline assessments were conducted in the hospital. The remaining follow-up assessments were also to be carried out in the hospital. However 2 months into the study it became clear that loss to follow-up was greater than expected. Of the 31 women who agreed to participate during this period, 18 (58%) failed to attend their scheduled follow-up appointments at 4 and 8 weeks. This was despite being offered transport to attend and reimbursement for their time and inconvenience. Women verbalized that attending the hospital was more disruptive than they had anticipated. As a consequence, and following discussion with the Data Monitoring Committee and the manufacturers of the assessment instruments, we decided to conduct future assessments in the home.

#### Transepidermal Water Loss (TEWL)

A closed chamber TEWL instrument was used to measure the flux of water vapour evaporating from the skin surface (AquaFlux Model AF200). The measurements were done by the same midwife at each time point for the same participant. The midwife was formally trained in obtaining such measurements, which were in accord with published guidelines for TEWL measurements [23]. Measurements were made twice at each of three sites. A baseline assessment was made prior to maternal transfer into the community and before first bath. A second assessment was made at 4 weeks and 8 weeks post birth. Measurements were taken on the upper abdomen (above nappy area), upper leg and forearm. The exact locations where measurements were performed were similar on all babies. This was achieved by measuring from anatomical markers such as skin crease of the wrist to midpoint on the volar forearm.

***Skin surface pH ***and ***hydration ***were measured at the same times and at the same sites as the TEWL measurements using a pH meter (Courage and Khazaka skin pH meter 900) and corneometer (Courage and Khazaka Corneometer CM 820).

#### Clinical observations

The skin was observed and recorded by the assessing midwife, at 4 and 8 weeks post birth using a validated rating scale which records erythema, dryness, scaling and need for medical products/attention [24]. Any skin treatments were recorded by the mother.

### Analysis

Data were input onto SPSS (Version 17) and double entered to ensure accuracy. In accordance with recommendations for pilot studies [25] data were summarized for the whole study group and tabulated according to allocation.

Individual experiences of women and members of the research team were recorded throughout the study to refine the study procedures for the main trial.

## Results

**Figure 1:**
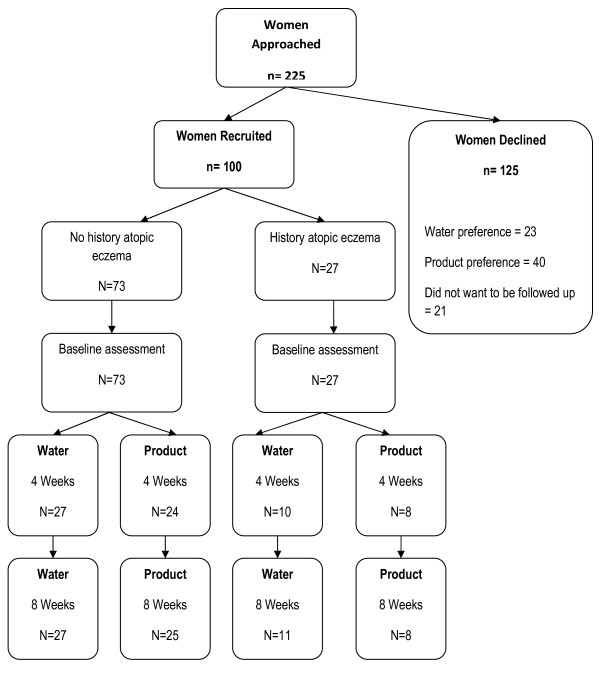
**Pilot study recruitment flow chart**.

Of 225 mothers who were approached to participate, 100 accepted. Figure 1 illustrates study recruitment, participant follow-up and reasons for declining. We did, however, conduct a post-hoc analysis according to assessment location. For all measures, we found no clear evidence of differences in reliability between locations on baby (arm/leg/abdomen) or place of assessment (home versus hospital). Reliability was good during hospital measurements and was maintained when we conducted follow-up assessments in the home. This was crucial to the success of the pilot, as our original plan to conduct all assessments in the hospital was unacceptable to women. The reliability of the tests was good. At all times and body locations the intra-class correlation of repeated measurements was at least 0.92, with an average difference of approximately 0.35, and most differences less than 2.0. Furthermore, the assessing midwife observed that babies being assessed at home were calmer than those in the hospital. Given the sensitivity of the TEWL instrument, it is therefore likely that more accurate readings were recorded as individual assessments were easier to take and position of repeat assessments was easier to locate. Figure 1 shows the number of assessments at home and in the hospital.

Table 1 illustrates the baseline details for the babies who participated. As shown, 27 participants had a family history of atopic eczema.

**Table 1:** Participant Baseline details

	No family history of atopic eczema *N *= 73		Family history of atopic eczema *N *= 27	
	**Water**	**Wash**	**Water**	**Wash**
	***N *= 37**	***N *= 36**	***N *= 14**	***N *= 13**

**Sex of baby**				
Male	15	17	6	5
Female	22	19	8	8

**Mums Ethnicity**				
White British	33	28	14	12
Black Minority Ethnic	0	4	0	0
Mixed Race	2	2	0	0
Other	2	0	0	0
Missing	0	2	0	1

**Baby's Ethnicity**				
White British	30	29	13	10
Black Minority Ethnic	0	2	0	0
Mixed Race	4	1	0	1
Other	1	0	0	0
Missing	2	4	1	2

**Feeding method**				
Breast	14	12	7	5
Bottle	21	21	7	8
Combined	2	3	0	0

**Parity**				
Primiparous	14	18	5	2
Multiparous	23	19	9	11

**Gestational age at birth**				
(days, mean (SD))	282.9 (6.3)	281.9 (7.3)	283.6 (7.2)	278.4 (6.7)

**Mode of birth**				
Caesarean section	0	0	0	0
Normal vaginal	33	34	14	13
Instrumental	4	2	0	0
**Maternal age **mean (SD)	26.4 (5.2)	27.2 (5.6)	29.2 (5.0)	29.8 (5.3)

An important reason for conducting the pilot was to determine compliance, in terms of the allocated trial arm and adherence to the bathing guidance. Compliance was shown to be an issue. Women's diaries and verbal reports indicated that between 3 and 4 weeks post birth, mothers perceived their baby's skin to be becoming dry. Although we requested that women refrain from using additional products on their babies' skin, this was the time in which they were most likely to introduce products into bathing regimes. As a consequence, there were 53 babies using products at the time when our primary outcome measure was being assessed; this was similar in each treatment group and despite the fact that women remained committed to completing the study. The number using products may in fact be an underestimate as some women may not have revealed the protocol violation. Women appeared to comply with the minimum bathing occasions of three; the median number of bathing occasions per week were 3 (range 2-7) for both groups.

As can be seen from table 2, there is no consistent evidence of numerical differences or trends in the data, between the trials arms, in either direction. This is true within all assessments (TEWL, hydration and skin surface pH) and location of assessments. Similarly, there is no evidence of difference between those babies with a family history of atopic eczema and those without. We calculated the 95% confidence intervals (CI) for the average TEWL measurement at each time point. At baseline the CI for the entire sample (*n *= 100) was 10.8 g/m2/h to 11.7 g/m2/h; after intervention at 4 weeks it was 10.9 g/m2/h to 13.3 g/m2/h (product) and 10.9 g/m2/h to 12.2 g/m2/h (Water); at 8 weeks it was 11.4 g/m2/h to 12.9 g/m2/h (product) and 11.4 g/m2/h to 12.9 g/m2/h (Water).

**Table 2:** Skin Functional Parameters/assessments

	No family history of atopic eczema *N *= 73		Family history of atopic eczema *N *= 27	
	**Water**	**Wash**	**Water**	**Wash**
	***N *= 37**	***N *= 36**	***N *= 14**	***N *= 13**

**TEWL **(g/m2/h)				
**<24 hours**				
Arm	12.7 (3.0)	12.2 (2.6)	11.8 (2.3)	11.8 (2.4)
Leg	12.0 (2.8)	11.1 (1.8)	10.9 (1.6)	11.5 (2.6)
Abdomen	10.4 (2.9)	10.4 (2.5)	9.6 (2.1)	9.2 (2.0)
**4 weeks post birth **Arm	12.1 (2.7)	12.6 (3.7)	12.1 (2.7)	12.8 (2.9)
Leg	12.2 (1.6)	12.5 (3.7)	12.2 (1.6)	14.3 (4.1)
Abdomen	10.1 (2.1)	10.7 (3.8)	10.1 (2.1)	11.2 (2.5)
**8 weeks post birth**				
Arm	11.1 (2.1)	12.5 (2.8)	11.1 (2.1)	13.1 (3.9)
Leg	11.9 (2.1)	12.6 (2.3)	11.9 (2.1)	12.7 (3.2)
Abdomen	11.9 (3.2)	11.3 (2.4)	11.9 (3.2)	11.4 (1.9)

**Hydrometer **(AU)**<24 hours**				
Arm	36.1 (8.2)	32.8 (7.8)	40.7 (10.7)	36.6 (11.8)
Leg	35.0 (9.8)	31.0 (7.2)	35.0 (10.0)	36.6 (9.8)
Abdomen	41.1 (13.6)	37.7 (8.4)	41.8 (9.7)	42.0 (8.3)
**4 weeks post birth**				
Arm	68.1 (11.3)	66.5 (13.3)	64.9 (14.7)	64.2 (13.9)
Leg	58.1 (14.5)	57.7 (11.3)	57.7 (13.7)	59.0 (13.2)
Abdomen	75.0 (10.7)	74.5 (9.4)	73.7 (16.4)	71.3 (11.6)
**8 weeks post birth**				
Arm	74.4 (12.8)	74.4 (11.2)	72.1 (13.0)	68.6 (15.9)
Leg	68.1 (12.0)	65.9 (13.8)	63.8 (9.4)	61.4 (16.0)
Abdomen	65.2 (12.4)	70.0 (10.9)	69.5 (13.1)	67.6 (10.4)

**Skin Ph <24 hours**				
Arm	6.89 (0.58)	6.76 (0.53)	6.63 (0.74)	6.71 (0.88)
Leg	6.91 (0.78)	6.69 (0.59)	6.44 (0.66)	6.58 (0.67)
Abdomen	6.90 (0.60)	6.63 (0.56)	6.76 (0.62)	6.80 (0.85)
**4 weeks post birth**				
Arm	5.06 (0.43)	5.17 (0.37)	5.01 (0.52)	5.19 (0.28)
Leg	5.14 (0.38)	5.31 (0.45)	5.07 (0.43)	5.20 (0.50)
Abdomen	5.29 (0.38)	5.30 (0.35)	4.92 (0.51)	5.47 (0.44)
**8 weeks post birth**				
Arm	5.14 (0.36)	5.12 (0.32)	5.13 (0.31)	5.09 (0.30)
Leg	5.11 (0.34)	5.27 (0.58)	5.01 (0.37)	5.24 (0.52)
Abdomen	5.27 (0.38)	5.40 (0.51)	5.05 (0.33)	5.27 (0.68)

The midwife assessed the babies' skin, according to a rating scale [24] at 4 and 8 weeks post birth. The rating scale contained three observations; dryness, erythema and breakdown/excoriation. Each observation was scored separately; a score of 1 indicated no evidence of abnormal skin whilst a score of 3 indicated some severity. None of the babies in the study scored 3, when assessed. As can be seen in table 3, few babies scored 2. The remainder of babies scored 1.

**Table 3:** Clinical skin assessment

	No family history of atopic eczema *N *= 73		Family history of atopic eczema *N *= 27	
**Skin assessment scale **(recorded by midwife)	Water *N *= 37	Wash *N *= 36	Water *N *= 14	Wash *N *= 13

**Baseline**				
**Dryness **(2 - Dry skin, visible flaking)	4 (4%)	6 (6%)	3 (3%)	3 (3%)

**4 weeks**				
**Dryness **(2 - Dry skin, visible flaking)	3 (4%)	5 (7%)	1 (1%)	1 (1%)
**Erythema **(2 - Visible erythema <50% of body surface)	3 (4%)	3 (4%)	2 (3%)	2 (3%)

**8 weeks**				
**Dryness **(2 - Dry skin, visible flaking)	1 (1%)	0	1 (1%)	0
**Erythema **(2 - Visible erythema <50% of body surface)	3 (4%)	6 (8%)	3 (4%)	2 (3%)

**Need for skin treatment**	1*	2*	0	0

* Health professional prescribed aqueous cream

## Discussion

The primary purpose of conducting this pilot trial was to inform a robust definitive trial of water and cotton wool versus a mild wash product for newborn babies. We present one of the largest baseline datasets on newborn skin assessments to date; information which is pivotal to the design of future studies in this field. However, when we set out to design the trial there was little published information on methodology or data from studies on newborns to assist in trial design. Although it is important to report what works in a study (as is usual in reports of a main trial), it is also important to share what does not work. There are many ways to design this type of trial and the field will only advance if the processes of trial design are shared transparently. In doing this, we reveal a number of important process issues, that would not normally be available to readers.

In our qualitative study [11], we asked women to indicate what they thought would be the optimum time to be approached about participating in this trial. Views were mixed; some suggested that the antenatal period was best, while others recommended the postnatal period. In this pilot study we decided to give women information in the antenatal period and re approach them in the postnatal period. The reality was that the majority of women had not absorbed the information prior to giving birth and/or was not ready to make a decision until the baby was born. It was only after giving birth to a healthy baby, and being faced with a real decision, that, for most women, the information was internalized and informed consent could be obtained. Although postnatal consent is appropriate, no woman objected to being given the information in the antenatal period, therefore the approach adopted was acceptable.

We conducted our pilot trial based on a superiority hypothesis. However, although the study was not designed to carry out a hypothesis test, examination of the data suggested that the hypothesis was not plausible. There was no convincing trend for superiority for any measurements on any part of the body. Furthermore, there was no clear evidence of any differences in any direction. The size of any of the small differences observed was deemed of little clinical importance by the Trial Steering Committee and Data Monitoring Committee. Therefore, a trial designed on the principles of non-inferiority appears most appropriate. The trial design therefore should be to generate data concerning the hypothesis that this mild skin cleansing product is not inferior to bathing with water only in its effect on skin barrier function. Moreover, as there was no difference between body parts, it seems reasonable, in future trials, to analyze an average assessment score.

There was no evidence of consistent differences between those babies with and without a family history of atopic eczema. If those with a family history of atopic eczema had shown more evidence of barrier dysfunction, this may have led us to design a trial based on this population only. Such a trial would be attractive because if a trial recruited a group with a propensity to disease and found no difference between treatments it would be unlikely that we would find a difference in a 'healthy' population. Given our results, however, it appears appropriate to include both groups, with stratification for family history. On a practical level this is more feasible, as those with a family history of atopic eczema were particularly difficult to recruit.

The importance of assessing compliance was highlighted in this pilot. Although women in our qualitative study [11] and at recruitment for this pilot study told us that they were happy to conform to protocol, compliance was an issue, making the findings difficult to interpret. This is one possible explanation for not observing a treatment difference. Some women, particularly those having their first baby, may not have been able to anticipate the difficulties of daily routines with a newborn baby. Given that parents introduced products around 3-4 weeks, and it is impossible to enforce or ensure compliance, it is more appropriate to have a primary endpoint prior to this.

This study provides an important exemplar of the importance of conducting a pilot study, particularly when there is a dearth of prior knowledge. The findings have indicated that the research processes, trial management and chosen primary outcome (i.e. TEWL) were appropriate. The feasibility of the main trial was also established. The trial management group and the independent Data Monitoring Committee have reviewed the process and data. A small number of important amendments to the trial have been made as a result of the findings of the pilot study. Our experience illustrates some contrasts between our qualitative study about the issues involved in a potential trial and what actually happened. For example the timing of information-giving was refined from the suggestions arising from the qualitative study. This provides an instructive example of the need to develop a large trial in several stages.

Three relevant RCT's, two by the same authors, were published after the completion of our pilot trial. Bartels study [26] tested the hypothesis that neither twice-weekly washing nor bathing would harm the natural adaptation of the skin barrier with respect to long-term effects on skin function in healthy newborns. The bathed group showed statistically significant lower TEWL on the buttock and higher hydration on abdomen and forehead compared to the wash group at day 28. The authors claim that both skin care regimes do not harm the adaptation of the skin barrier in healthy newborns in the first 24 hours of life. The second study [27], aimed to test the hypothesis that twice-weekly bathing with a commercially available baby wash gel and additional baby cream would not harm the natural adaptation of skin barrier in healthy newborns. At 8 weeks, the group using clear water and topical cream had lower TEWL measurements on their fronts, abdomen and upper legs as well as higher stratum corneum hydration on their fronts and abdomen compared with bathing with water only. The group bathing in wash gel had lower pH on all sites compared with bathing in water only at week 8. No differences in sebum levels, microbiological colonization and skin scores were found. The authors conclude that skin adaption as a barrier function was not harmed by tested skincare regimens in full term healthy infants. The final RCT [28] was a three armed trial, conducted in the Philippines, which compared a Johnson's^® ^*baby top- to-toe™ wash *(Johnson & Johnson Consumer Companies, Inc.) with Sebamed^® ^baby liquid cleanser and water alone; 60 babies were randomized in each arm. Assessment measures were similar to those reported in the Garcia Bartels studies, with the addition of skin oxyhemoglobin, deoxyhemoglobin and parental satisfaction measures. The authors conclude that all three regimes are 'safe for use in infants with normal skin.' However, although these three studies provide novel information relating to term baby bathing none report a priori primary outcomes or sample sizes, which make it difficult to assess the extent to which the results could have arisen by chance. Furthermore, it is not clear how the results of either trial relate to clinically important safety outcomes since the investigators do not state what they mean by "harm". None of these published studies use home measurements. In our experience the families who complete a trial using hospital assessments are a subset of families who were committed to the study. This subset may not be representative. Such a committed group may be particularly concerned about skin and skin care. This concern may mean that their skin care practices at home may be different from other families. We have described and validated an approach that reduces the potential for selection bias.

## Conclusion

Our study adds to existing literature by providing valuable baseline data and important information on trial processes. Our study observations were consistent with previously published papers but we believe that the way forward is to test the hypothesis in a properly designed and adequately powered non-inferiority trial.

## Competing interests

This study was funded by Johnson and Johnson. However, the study was investigator led. TL, CB and MC have acted as temporary advisors to J & J previously.

## Authors' contributions

TL and MC conceived the idea. TL, AH, MC, MT, CB and EO designed the study. CB and EO collected the data. AH, TL, CB and EO analyzed the data. TL and AH wrote the original draft of the paper. All authors commented on the paper and agreed the final version.

## Pre-publication history

The pre-publication history for this paper can be accessed here:

http://www.biomedcentral.com/1471-2431/11/35/prepub
